# Service user and family member perspectives on services for mental health, substance use/addiction, and violence: a qualitative study of their goals, experiences and recommendations

**DOI:** 10.1186/s13033-016-0040-3

**Published:** 2016-02-20

**Authors:** Rebecca Haskell, Kathryn Graham, Sharon Bernards, Andrea Flynn, Samantha Wells

**Affiliations:** Social and Epidemiological Research Department, Centre for Addiction and Mental Health, 200-100 Collip Circle, London, ON N6G 4X8 Canada; Department of Psychology, Western University, London, ON Canada; Dalla Lana School of Public Health, University of Toronto, Toronto, ON Canada; School of Psychology, Faculty of Health, Deakin University, Geelong, VIC Australia; National Drug Research Institute, Curtin University, Perth, WA Australia; Provincial System Support Program, Centre for Addiction and Mental Health, London, ON Canada; Department of Epidemiology and Biostatistics, Western University, London, ON Canada

**Keywords:** Mental health, Substance use/addiction, Violence, Qualitative, Interviews, Individuals who use services, Families, Caregivers

## Abstract

**Background:**

Mental health and substance use disorders (MSD) are significant public health concerns that often co-occur with violence. To improve services that address MSD and violence [MSD(V)], it is critical to understand the perspectives of those most affected, people who have sought help for MSD(V) (i.e., “service users”), especially those with co-occurring issues, as well as their family members.

**Methods:**

We conducted structured interviews with 73 service users and 41 family members of service users in two Ontario communities (one urban, one rural) regarding their goals related to help-seeking, positive and negative experiences, and recommendations for improving systems of care.

**Results:**

Overall, participants expressed a need for services that: (1) are respectful, nonjudgmental, and supportive, help service users to feel more ‘normal’ and include education to reduce stigma; (2) are accessible, varied and publicly funded, thereby meeting individual needs and addressing equity concerns at a systems level; and (3) are coordinated, holistic and inclusive of family members who often support service users.

**Conclusions:**

The findings provide a rich understanding of how service users and their families perceive services for MSD(V) issues and identify key ways to better meet their needs.

## Background

Mental and substance use disorders (MSD) are significant public health concerns that impose considerable economic, health and social costs [1, 2]. For example, the WHO Global Status Report on Alcohol [2] indicated that about 5.1 % of the global burden of disease and injury was attributable to alcohol use. According to the 2010 Global Burden of Disease study, MSD accounted for 183.9 million disability adjusted life years (DALYs) or 7.4 % of all DALYs worldwide [1]. In 2011, it was estimated that over the 20 subsequent years, costs due to mental illness will amount to US$16 trillion worldwide [3].

Mental health challenges often co-occur with substance use issues [4, 5]. These issues also commonly intersect with experiences of trauma and violence; that is, although most people who have mental health/substance use issues are not violent, evidence shows higher rates of past and present abuse and trauma in their lives [6] as well as current or on-going partner and non-partner aggression both as victims and perpetrators, compared with individuals who do not have mental health [7], or substance use [8, 9] issues. Evidence also indicates that both victims and perpetrators of spousal abuse have poorer mental health than do those who do not experience partner violence [10–12]. The co-occurrence of mental health, substance use, and violence issues [MSD(V)] creates service needs that are complex [13] and require comprehensive and coordinated care [5, 14]. To align services with the needs of individuals who face MSD(V) issues, especially those who have concurrent issues, researchers and practitioners require a better understanding of the experiences and perspectives of people who have sought help for MSD(V) [15] as well as the perspectives of their family members [16].

There is a growing emphasis in the field on mental health “recovery” which is defined as “a way of living a satisfying, hopeful, and contributing life even with any limitations caused by illness” [17]. Based on the perspectives of individuals with lived experience, a recovery-oriented model highlights the importance of hope, self-determination, agency, meaning/purpose, and awareness/potentiality [18]. Consistent with this, desirable recovery-oriented outcomes and services reported by individuals using mental health services in nine of the Unites States included: supporting basic material needs; fostering opportunities to engage in social relationships and meaningful citizenship activities; and attending to their overall ‘personhood’ [19].

Research on the subjective experiences of individuals who use MSD(V) services shows that they place a great deal of importance on being treated with respect, dignity and being listened to. For example, Gumber and Stein [20] found that accepting attitudes and a willingness to share information with clients were key qualities of services that were perceived as helpful by persons with schizophrenia. Studies of women who used alcohol and drugs [21] and those who had co-occurring MSD(V) issues [22, 23] found that they appreciated interactions with service providers in which they were listened to and treated respectfully, and where their expertise regarding their own health was recognized. Programs and services that acknowledge and attend to the contexts of people’s lives have also been identified as being important, including understanding and respecting individuals’ ethnic and cultural backgrounds [24, 25].

Existing research also indicates a need for services that better address intersecting mental health and substance use needs, continuity of care, and assistance navigating health systems. Prior research with people who have MSD issues has found that service providers should be prepared to serve people with complex needs [26, 27]. This complexity is exacerbated when individuals face issues related to violence in addition to MSD issues. For example, women facing violence/trauma as well as MSD recommended the provision of a caring and safe environment and programming that takes into account both abuse and MSD-related factors [22, 23]. Research also indicates that individuals who use MSD(V) services stress the need for continuity of care to address co-occurring issues through better service integration and coordination between different service providers. Additionally, stronger links between health and social services are emphasized as being important, so that individuals’ practical needs are met (e.g., housing, prenatal or child care, income, employment, and transportation), which are a foundation for mental well-being [26, 27].

Previous research on the needs of persons with MSD(V) issues has tended to focus mostly on specific sub-populations of people with mental health issues (e.g., schizophrenia), specific experiences of violence (e.g., violence against women) or on people who have sought help at specific agencies (e.g., domestic violence shelter or short-stay substance use service). As such, the findings may not reflect the heterogeneity and complexity of the issues people face or the range of people’s experiences with the system of services. To better meet the needs of heterogeneous populations of people with MSD(V) issues, research is needed on the experiences and perspectives of individuals who cope with a range of MSD(V) challenges, especially those with complex needs. In addition, previous research on people’s experiences has focused mainly on negative components, such as barriers to receiving care and unmet need, with less attention paid to identifying positive aspects of the system [13]. Understanding both positive and negative experiences is essential for identifying ways that the system of care can be improved.

In sum, there is a need for qualitative research from the perspective of individuals who use MSD(V) services that can provide important knowledge regarding strengths and gaps in the system of services and how these contribute to the recovery process [15, 16, 28]. Including the perspectives of family members, who often facilitate help-seeking and provide other supports to service users [29, 30], can also provide an important perspective on the system of services for people with MSD(V) issues and on flaws and strengths in the system [31].

Drawing on the positive and negative experiences of individuals who use MSD(V) services and family members of service users, this research aims to identify ways to better meet the needs of persons with MSD(V) issues by clarifying:

1. what they hope to gain when they or their relatives seek help, and
2. ways that services and the system generally can better meet their needs or those of their family members.

## Methods

The present study was part of a larger project that used mixed methods to collect data on MSD(V) issues in diverse communities, including urban, rural and disadvantaged communities, using a mobile research laboratory [32]. This article describes findings from a structured interview study of people’s experiences seeking/receiving help for MSD(V) issues in two communities (one rural, one urban) in Southwestern Ontario. We chose a qualitative approach to the research to capture the complexity of issues and to be sensitive to the highly personal and sometimes emotional nature of experiences [33].

### Sampling and recruitment

We used purposive sampling to recruit individuals who used MSD(V) services and family members who could help to answer our research questions [34]. Based on a literature review and in collaboration with a local Community Advisory Committee, eligibility criteria included the following: 18 years or older, resident of the local community, and self-identified as having personally experienced a mental health or substance use problem and sought help for that problem within the previous 5 years (“Service User” sample) or had a relative with previous or current problems related to mental health or substance use who had sought help for these problems within the previous 5 years (“Family Member” sample). Thus, although many family members were currently or had been in a caretaking role of a family member with MSD issues, not all family members were necessarily directly involved in their care. The two samples were independent; that is, no family members were related to participants in the service user sample. The 5-year timeframe helped to ensure that participants were able to share relatively recent experiences with existing services and to reduce recall problems. Experiences of violence were not necessary for eligibility, but were captured as part of the interview process.

To recruit a sample of service users with a wide range of mental health, substance use and violence issues, including co-occurring issues, posters and flyers were placed in mental health, addiction, and violence agencies, social service agencies, community organizations, public locations (e.g., libraries, coffee shops, community bulletin boards), and in local newspapers (both print copies and online). Similar posters were also displayed to recruit family members of service users. The posters and flyers indicated that participants would be compensated for their time but not the amount. We also presented information about the study to service providers who shared this information with clients or family members of clients.

To enquire about participating in the study, individuals contacted the interview coordinator by telephone, text message, email or by going to the mobile lab during specified drop-in hours.

The interview coordinator provided all interested persons with information about the study, screened them for eligibility, and scheduled appointments with those eligible. The interview coordinator was instructed to include in the study anyone who perceived they had a mental health or substance use issue and had made any effort to receive treatment or support from formal or informal services within the region for an MSD issue (if they had received treatment/support outside the region they had to be a resident of the region). Eligible participants were advised at this time that they would receive a $25 gift card for their participation. Those who were not eligible or chose not to participate were offered a resource package containing information about local services for MSD(V) issues.

Participants were recruited until “data saturation” was reached, that is, sufficient data had been collected to ensure replication and no new data were surfacing from the interviews (see “Analyses” section below) [35]. A large sample size was needed to capture variability in people’s lived experiences and constellations of MSD(V)-related issues from the perspective of both service users and family members and rural and urban residents. A total of 114 participants were interviewed in the two communities (73 service users; 41 family members). About half (51 %) of service users and most (83 %) family members were women; 77 % of service users and 76 % of family members were from the urban community. Additional demographic information regarding participants is provided in Table 1.

**Table 1:** Characteristics of participants in the service user and family member studies

	Service users (*N* = 73)	Family members (*N* = 41)	All (*N* = 114)
	*N* (%)	*N* (%)	*N* (%)
Community			
Urban community	56 (76.7 %)	31 (75.6 %)	87 (76.3 %)
Rural community	17 (23.3 %)	10 (24.4 %)	27 (23.7 %)
Gender^a^			
Men	36 (49.3 %)	7 (17.1 %)	43 (37.7 %)
Women	37 (50.7 %)	34 (82.9 %)	71 (62.3 %)
Age group^b^			
18–24	7 (9.7 %)	4 (10.8 %)	11 (10.1 %)
25–34	11 (15.3 %)	5 (13.5 %)	16 (14.7 %)
35–49	26 (34.1 %)	10 (27.0 %)	36 (33.0 %)
50 and older	28 (38.9 %)	18 (48.6 %)	46 (42.2 %)
Education level^c^			
Less than high school	18 (25.0 %)	5 (13.9 %)	23 (21.3 %)
High school	15 (20.8 %)	9 (25.0 %)	24 (22.2 %)
Post-secondary	39 (54.2 %)	22 (61.1 %)	61 (56.5 %)
Employment status^b^			
Working for pay/self-employed	9 (12.5 %0	11 (30.6 %)	20 (18.5 %)
Unemployed	21 (29.2 %)	7 (19.4 %)	28 (25.9 %)
Long term illness or disability	32 (44.4 %)	13 (36.1 %)	45 (41.7 %
Student or retired	10 (13.9 %)	5 (13.9 %)	15 (13.9 %)
Marital status^b^			
Never married	27 (37.5 %)	9 (25.0 %)	36 (33.3 %)
Married	9 (12.5 %)	7 (19.4 %)	16 (14.8 %)
Common-law	8 (11.1 %)	7 (19.4 %)	15 (13.9 %)
Separated/divorced	25 (34.7 %)	12 (33.3 %)	37 (34.3 %)
Widowed	3 (4.2 %)	1 (2.8 %)	4 (3.7 %)

^a^No participants in this sample identified as transgender, although that option was available

^b^Missing data for five participants

^c^Missing data for six participants

To assess source of recruitment, participants were asked an open-ended question about how they learned about the study. Of those who provided responses (89 % of service users and 81 % family members), most (60 % service users, 39 % family members) learned about the study from a poster or flyer placed in community agencies (11 different agencies were mentioned by service users, and 7 different agencies were mentioned by family members) and other public locations. Participants also learned about the study through their peer networks such as a friend or family member (22 % service users, 30 % family members). Other participants dropped in at the mobile lab to enquire about the project (5 % service users, 12 % family members), or found out about the study from a newspaper or radio advertisement (3 % service users, 12 % family members), a worker or staff member at a MSD(V) agency (6 % service users, 3 % family members) or a presentation by the interview coordinator (4 % service users, 3 % family members). Overall, the sample was recruited through a wide range of sources.

### Procedures

Interviews were held in a private area where information was provided about the study including potential risks and benefits, confidentiality, and assurance that participants could refrain from answering any questions or end the interview at any time. After reviewing this information, all participants provided written consent and gave permission for their interviews to be audio-recorded. The interview script was developed based on the literature, our research questions and input from the local Community Advisory Committee. Questions focused mainly on the experiences of the service user—from the service user’s own perspective and from the perspective of the family member. Participants were first asked about the types of issues they had experienced, including mental health, substance use/addiction, and violence. Specific questions and probes related to the content included in the present analyses are listed in Table 2. These questions focus on desired goals and outcomes and ways to improve systems of care based on positive and negative experiences getting help. As noted in the table, additional probes were provided to explore the issue more fully with the participant when necessary. Interviews ranged from 30 to 90 min and participants were given their choice of a $25 gift card to a local grocery store, pharmacy or coffee shop in appreciation for their participation. They were also given a resource package containing information about local services for MSD(V) issues. All interview recordings were transcribed for analyses.

**Table 2:** Interview questions analyzed in this article

Consumers	Family members
Desired goals and outcomes	
What did you want to achieve/what were your goals when you went for help for the MSV problem(s)? Probes included: What would be the ideal/best outcome for you? What would be an okay outcome? What would be the worst outcome?	To your knowledge, what did your family member want to achieve/what were their goals when they went for help for their mental health, substance use or violence? Probes included: What would be the ideal/best outcome for your family member from their perspective? What would be an okay outcome for your family member from their perspective? What would be the worst outcome for your family member from their perspective?
	What about your goals for your family member? Probes included: What would be the ideal/best outcome for your family member from your perspective? What would be an okay outcome for your family member from your perspective? What would be the worst outcome for your family member from your perspective?
Ways to improve the system based on positive and negative experiences	
Was there anything that you found especially helpful or that made getting help easier or better for you? What made it easier?	Was there anything your family member found especially helpful or that made getting help easier/better for them? What made it easier?
What were the best experiences you had while you were getting help for MSV problems?	What were the best experiences your family member had while they were getting help for MSV problems?
Was there ever a time when you didn’t get the help you wanted? What happened?	Was there ever a time when your family member didn’t get the help they wanted? What happened?
Have there been times you felt that your needs weren’t all met? What needs weren’t met? What might have been done to better meet your needs?	Have there been times when they felt that they did not have all of their needs met? What were these needs? What might have been done to better meet their needs?
	Were there times when *you* felt that they didn’t have all of their needs met? What were these needs? What do you think might have been done to better meet your needs?
Was there anything that happened while you were getting help that made the problems worse or that made recovery more difficult? What happened?	Was there anything that happened while your family member was getting help that made their problems worse or made their recovery more difficult? What happened?
What do you think are the main challenges/barriers that you faced trying to get help for the problems?	What do you think are the main barriers/challenges that your family member faced in trying to receive help for their problems?
Based on your own experiences, what suggestions do you have for ways to improve services or supports for people who have MSV problems?	Based on how things have went for your family member and the impact that their experiences had on you and your family, what suggestions do you have for ways to improve services for people who have mental health, substance use or violence problems?

### Analyses

We used a grounded theory approach [36] with data collection and initial analysis occurring simultaneously. Throughout the research and analysis, codes encompassing emerging patterns and themes were developed, revised and used to organize and classify data. For the initial round of interviews, we identified initial themes and developed data coding sheets. Although the content and open-ended format of the interview remained unchanged, the coding sheets were used during subsequent interviews to categorize open-ended responses. Notably, response codes were completed by the interviewer and not presented to the participant. After data collection, all interview transcripts were carefully analyzed, with existing themes refined and additional themes identified. Analyses continued until no new themes were identified within the specific set of questions we analyzed and all themes were fully described. In this way, the emergent themes are grounded in the data and are not the result of, nor measured against, an initial hypothesis or preconceived theory.

In the present paper, excerpts from the interview transcripts are used to illustrate the emergent research themes and common experiences reported by participants. As part of the analysis, we explored similarities and differences between the perspectives of service users and family members and those who lived in rural versus urban settings. Due to the smaller number of participants from the rural community and because both communities are part of the same health planning area, data from both communities were combined in the analysis; however, we note where findings are particularly relevant to one community. Similarly, themes for service users and family members are presented separately only when these views differ.

### Ethics, consent and permissions

This study was approved by the Research Ethics Board of the Centre for Addiction and Mental Health. All participants provided written consent to participate in the study. Quotes from participants were edited to eliminate identifying information.

## Results

Service users and family members were asked to identify the types of problems they or their relative experienced. As shown in Table 3 for service users, family members, and the total sample, about 84 % of participants (85 % of service users and 83 % of family members) reported a mental health issue, 70 % (69 % of service users, 73 % of family members) reported a substance use/addiction issue and about 66 % (66 % of both service users and family members) reported any anger, abuse or violence/aggression as a child or as an adult. Overall, about 80 % of participants reported they or their family member experienced co-occurring issues (78 % service users, 84 % family members) with 40 % describing issues in all three areas; conversely, few had only one issue (15 % mental health only, 5 % substance use/addiction only, none violence/aggression only).

**Table 3:** Types of MSD(V) issues reported by service users and family members

Type of issues	Service users (N = 73)	Family members (N = 41)	All (N = 114)
	N (%)	N (%)	N (%)
Mental health	62 (84.9 %)	34 (82.9 %)	92 (84.2 %)
Depression/suicidal	44 (60.3 %)	21 (51.2 %)	65 (57.0 %)
Anxiety/panic disorders	29 (39.7 %)	8 (19.5 %)	37 (32.5 %)
Bipolar/manic	24 (32.9 %)	13 (31.7 %)	37 (32.5 %)
Schizophrenia	2 (2.8 %)	6 (14.6 %)	8 (7.0 %)
Other mental health issues (e.g., ADD, ADHD, sleeping disorder, eating disorder, gambling, stress)	18 (24.7 %)	14 (34.1 %)	32 (28.1 %)
Substance use/addiction	50 (68.5 %)	30 (73.2 %)	80 (70.2 %)
Alcohol	30 (41.1 %)	16 (39.0 %)	46 (40.4 %)
Drugs	45 (61.6 %)	23 (56.1 %)	68 (59.6 %)
Anger, abuse, violence or physical aggression	48 (65.8 %)	27 (65.9 %)	75 (65.8 %)
Anger	9 (12.3 %)	2 (4.9 %)	11 (9.6 %)
Adult abuse/violence	8 (11.0 %)	3 (7.3 %)	11 (9.6 %)
Trauma	4 (5.5 %)	2 (4.9 %)	6 (5.3 %)
Childhood physical abuse/violence	2 (2.7 %)	1 (2.4 %)	3 (2.6 %)
Childhood sexual abuse/violence	2 (2.7 %)	1 (2.4 %)	3 (2.6 %)
Physical aggression in past 5 years:	44 (60.3 %)	22 (53.7 %)	66 (57.9 %)
As victim only (toward service user)	20 (27.4 %)	3 (7.3 %)	23 (20.2 %)
As perpetrator only (by service user)	3 (4.1 %)	6 (14.6 %)	9 (7.9 %)
As victim and perpetrator	21 (28.8 %)	13 (31.7 %)	34 (29.8 %)

Percentages do not add to 100 % because some participants described multiple issues

In the following, we describe the main themes to emerge in relation to our two broad research questions (1) desired goals and outcomes when seeking help, and (2) ways to improve services/systems. The second theme (ways to improve services/systems) was further categorized in terms of: (a) types of services and how they are provided, (b) broad system of care, and (c) system issues specifically of concern to family members (see Table 4 for a listing of all main themes).

**Table 4:** Main themes identified from service user and family member interviews

*Desired Outcomes When Seeking Help*
Having MSD(V) issues improve
Understanding MSD(V) issues better
Feeling/being “normal”
Addressing practical needs
Improving social relationships
*Ways to Improve Services/Systems*
(a) Types of services and how they are provided
Being listened to, not judged and treated with respect
Availability of peer support and help from people who have lived experience
Appropriate use of medications and related support
Recreation activities
Assistance with practical needs
(b) Broad system of care
Coordinated holistic care and help navigating the system
More accessible publicly funded services
Early intervention
(c) System issues specifically of concern to family members
A system that supports greater involvement of family members
Mechanisms for treatment compliance

### Desired outcomes when seeking help

Participants were asked what they wanted for themselves or their family members when seeking help for MSD(V) issues. As shown in Table 4, the main themes for both service users and family members included a range of outcomes from having MSD(V) issues improve to improving social relationships. Illustrative quotes for each of these themes are marked “SU” if reported by a service user and “FM” if reported by a family member.

#### Having MSD(V) issues improve

Many participants indicated that they or their family member would likely deal with the MSD(V) issues their entire life. Nevertheless, regardless of the type of issues they experienced, participants hoped that the service user’s issues would improve after receiving care, including reducing or stopping substance use, better coping with mental health symptoms and, ultimately, being happier, less anxious and more relaxed. Asked what they wanted to achieve when seeking help for MSD(V) issues, service users said, for example: “I know that I will never be cured from depression. I’m going to have it my whole life. My goal is to handle it a little bit better” (SU); “just to be able to function in society” (SU); “total abstinence” (SU); “I wanted to get off the drugs” (SU).

Improvements in co-occurring issues and multiple life areas were frequently mentioned: “for the violence I was looking for a safe place to go where I can feel safe and as for the addictions, I was looking to get some level of sobriety” (SU); “get his anger under control and his substance abuse under control and [get] the proper medication he needs for his mental issues and [get] properly diagnosed” (FM).

#### Understanding MSD(V) issues better

Some participants, especially service users, wanted knowledge and tools to better understand their issues and to cope with potential triggers and symptoms: “get my mental health and my pain down to a low roar where I could cope, you know, with understanding it” (SU); “help me understand…why I felt the way that I was feeling. How I could stop feeling that way” (SU).

#### Feeling/being “normal”

Many participants expressed the hope that supports would help them or their family members feel “normal;” that is, for service users to feel the way they did before they experienced MSD(V) issues or to be like others who do not have such issues. Related to this theme, service users expressed the following goals: “to have me back” (SU); “to be like a normal person…without anger, without drugs, without alcohol” (SU); “They wanted what they called a normal…life back” (FM); “to be clean from all the drugs, all the alcohol and all the violence…and [settle] down and [be] his old self” (FM).

An important part of the desire to be ‘normal’ included being able to contribute to society, for example, through employment, returning to school or volunteering: “it would be nice to go back to work” (SU); “getting back into school for sure” (SU); “I would really like to see him have a structure in place so that he had something productive to do to contribute…I don’t know how anybody…whether they have a mental illness or not could ever feel good about yourself by having nothing to do” (FM).

#### Addressing practical needs

Participants, especially family members, hoped that MSD(V) programs would assist service users to meet their basic and practical needs: “I would like for someone to recognize that she needs to be in a home that’s assisted…she can’t live on her own, [she needs] a rest home or a group home” (FM). Others wanted the service user to be more self-sufficient as a result of accessing support for MSD(V) issues: “get a job, put his life together, be independent, not needing me all the time, because it’s really over-taxing” (FM).

#### Improving social relationships

Family members wanted their relatives to be more caring and loving as a result of accessing services for their MSD(V) issues: “I wanted him to learn to love me” (FM); “that he would be able to achieve intimacy in his life with women, with myself and friends and with men—to not feel so threatened by that” (FM).

Both service users and family members expressed the importance of the service user ending relationships with people who had a negative influence and fostering more healthy relationships, particularly with family members: “the best thing that would happen [after getting help is]…to see my kids” (SU); “hopefully she would get rid of all these bad people around her, realize that they are not…helping her they are just, you know, destroying her life” (FM); “he has [his children] back in his life, and now he’s starting to build a wonderful relationship with his [grandchildren] that weren’t in his life for that length of time” (FM).

### Ways to improve services/systems

Ways that services and the system can better meet service users’ needs were drawn from participants’ descriptions of their positive and negative experiences getting help and suggestions for improving the system. As shown in Table 4, these are grouped under three subheadings, types of services and how they are provided, broad system of care, and system issues of special concern to family members.

#### Types of services and how they are provided

As shown in Table 4, themes pertaining to types of services and how they are provided range from the need to be listened to, not judged and treated with respect, to a need for recreation activities.

##### Being listened to, not judged and treated with respect

Service users appreciated having opportunities to talk about their MSD(V) issues, being listened to, taken seriously and treated with respect: “I just think that people need to listen and believe and treat them like they are important…just be respectful to them” (SU). Service users also described the importance of services and those providing support being welcoming, caring, understanding, non-judgmental and helpful. Speaking about a friend who had helped him, one service user said: “She just listens when I need to talk to somebody—she doesn’t judge me…she gives me pointers or tips of how I can do things differently” (SU).

Conversely, negative experiences involved being unable to access someone with whom service users could talk and work through their issues. Being disrespected was also noted, especially related to emergency department personnel and police: “because of the [substance use]…I’ve had seizures…I was there for 5 h in the ER and [the doctor] wouldn’t even come see me, wouldn’t give me any medications to help me to stop the seizure…I’d say…30 % of the doctors there…don’t look at you because of your addiction” (SU). About the police response to partner violence, a service user said: “I clearly had been abused…they just said it was all my fault because I had been drinking…it was very…demeaning and scary” (SU).

Service users also reported negative experiences with other health professionals: “[the psychiatrist] called me stupid, he used the word ‘stupid’. I was like wow, [the] whole point of me coming here is to get to the bottom of this” (SU). To avoid negative judgment from service providers, some service users noted that they withheld information about co-occurring MSD(V) issues; for example, asked whether staff at a local detox program enquired about issues outside of their addiction, a service user responded: “[they asked] ‘have you ever tried to kill yourself?’ Even though I have, I would say no. I just didn’t want to be labeled” (SU).

Participants attributed unhelpful responses to a lack of understanding of MSD(V) issues, stigma or a lack of time among service providers: “I find they [service providers] treat you a lot differently when they find out you have a mental illness…I think they should be educated more because a lot of people are scared of what they don’t know” (SU); “who wants to have the diagnosis that you have a mental illness? It’s just stigmatized…I’m aware of it because it happened in my family but if it wouldn’t have…I probably wouldn’t be at the level I’m at understanding mental illness either” (FM); “it’s like everything else, it depends on the people who run the program, it depends on who your worker is and if you happen to be the lucky one that gets a committed worker that’s really on the ball, great, but then that committed worker is also overloaded with cases they don’t have the time…” (FM).

To counter stigma and lack of awareness, many participants recommended outreach and education initiatives that would support helping professionals and the general public to develop a better understanding of MSD(V) issues and increase awareness of local services for these issues: “public awareness and education…they have those little commercials for blood pressure pills or whatever, they should have little commercials about mental health” (SU); “education, if we can educate not only ourselves but the public…then it’s just easier. I mean if I can say to somebody ‘I’m bipolar’ and they don’t snicker, or they don’t, you know, step aside because they think I’m going to hurt them or I’m going to go crazy on them” (SU).

##### Availability of peer support and help from people who have lived experience

Participants spoke positively about experiences where service users were able to discuss issues with and learn from peers or counsellors who had experienced MSD(V) issues: “it was really good to be able to talk with other people who are in the same situation” (SU); “the one counsellor was a recovered substance abuser himself…and he could relate to everything that they talked about. He had a lot of credibility with my son, because he had walked that path” (FM). Others recommended that programs hire peer support staff based on their experiences: “if you haven’t been through it you don’t understand, you can’t say ‘oh yeah, I understand’ and mean it” (SU). On the other hand, some service users felt peer support could trigger problems: “I had to sit there and…talk about drugs and stuff and just triggered my mind even more” (SU). Being in close proximity to other people with addictions was also described as a trigger for some, particularly in unstructured programs: “but I couldn’t stand that program. It was too much drugs around…for me, I am still too weak you know” (SU); “I could find better drugs at the NA meetings than I could in the street” (SU).

##### Appropriate use of medications and related support

The benefits of medications were mentioned by some participants: “I was on the right medication…I was getting the right help instead of just being on the street again and doing absolutely nothing but crime and drugs” (SU); “I’m on a low dosage [of methadone], so that’s good…I’m not nodding out, I just, I feel normal, I feel sober” (SU); “I think he has a chemical imbalance and the pills that he takes balance the chemicals in his brain” (FM).

However, some service users and family members felt that health care professionals were too quick to “push pills” without talking with the service user about their issues or considering the service user’s concerns about medications, including unwanted side effects or their addictive nature: “I went to [the doctor] before for depression a long time ago and he put me on Prozac, but it did the opposite…it made me suicidal…so I stopped taking them, but I’ve been suffering like forever” (SU); “he gave me drugs that were addictive” (SU); “when you [the doctor] are faced with a frustrating case, shouldn’t something like a little light go off in your head to tell you that maybe in this case I should look a little bit deeper, you know, instead of just concluding that this kid’s a head case and doesn’t want to take any medication” (FM)?

Participants suggested that medications are needed but only as one component of treatment: “psychiatrists should be able to talk to us as well, instead of just pushing pills down our throat” (SU); “why are they depressed? That’s what you should look into…That’s how they have to help. Ask questions, find out what is ailing them and heal them” (SU); “I honestly think the majority of times people need to talk their problems out. They don’t need to be medicated” (FM).

##### Recreation activities

In addition to having someone to talk to about their MSD(V) challenges, some service users spoke positively about programs with diverse recreation and learning activities as well as opportunities to socialize and talk to others: “there’s something for everybody. If you want to just socialize there’s that…and it keeps me out of bed, it keeps me going” (SU); “she’s doing a program now, it’s art therapy…it’s therapeutic, it helps them stop spinning…like the talking and anxiety, I think it helps” (FM).

##### Assistance with practical needs

Consistent with the desired outcome of having their practical needs addressed, participants described the importance of having access to services that provide advocacy, accompaniment to appointments, and assistance in meeting practical needs such as housing, financial needs and transportation (especially in the rural area): “even little things if I’m looking for a job or whatever, she’ll help me do that” (SU).

#### Broad system of care

As listed in Table 4, themes related to the broad system of care for MSV(D) issues included issues of coordination, access, funding and early intervention.

##### Coordinated holistic care and help navigating the system

Participants appreciated experiences at the program level in which the connections between the different issues they were facing were recognized and addressed. For example, when asked whether a doctor specializing in addictions enquired about problems with mental health or violence, a service user said: “he [the doctor] says ‘you are using drugs because of your mental health, because you are so depressed’…and I told him what my dad used to do to me. He said, ‘well you’ve got a lot of problems’…he wanted me to see my psychiatrist more” (SU). However, positive experiences with services that provided well-coordinated and holistic care were rare. Many participants, especially people who had co-occurring disorders, experienced challenges seeking help for MSD(V) issues due to the compartmentalized nature of the system of care for these issues. Participants reported difficulty getting help for more than one issue at a time and complained about having to repeat their story: “for me the experience has been tough because I am dealing with multiple different things…whether it’s sexual abuse, whether it’s also the alcohol and drug abuse, and then also the biological factors. So it’s a lot of different little pies and it seems like…you talk to this person for this section, and then you talk to another person for this section and…it doesn’t feel organized…and sometimes honestly they contradict each other” (SU); “you kind of get a little bit tired repeating yourself but they are supposed to be directing you to the right area so…I [worked] very hard to forget my life or not to deal with it so having to repeat it for multiple different people is a little frustrating” (SU); “he was at [the program] for 3 months…No one ever said to me that they thought that maybe he had an underlying [mental health issue], not any of the counsellors, not the director, nobody. So it was only focused on addictions” (FM).

Participants suggested that services need to be coordinated and holistic, with flexibility to meet individual needs: “gotta change the old hospital into a place where just people who have mental issues and then maybe on the other side drug issues…not just alcohol and drugs, I think even [eating disorders] because it has to do with some kind of emotional…I think if it was all in one place it would do a lot of good” (SU). Participants also suggested that service providers need to ask about issues that fall outside of a program’s primary mandate and be aware of and willing to make referrals to complementary programming: “do better assessments in identifying these issues instead of putting people in programs that kind of focus too much on one because…it’s all interrelated…I mean the approach should be more collaborative…and team oriented” (SU).

Participants especially recommended holistic services at the first point of contact, such as emergency department settings and contacts with the police: “the initial reach for help, like for example emergency room, to have available staff that are educated on substance abuse and mental health and that’ll assess appropriately in terms of—is there a situation of violence that’s going on? Like to be aware and to have referrals available that’s appropriate” (SU); “so that you’re not going to have any bully police officer there who doesn’t understand mental illness—you’re going to have someone who’s trained to know what to say to kind of assess the info, the situation and be helpful” (FM).

##### More accessible publicly funded services

Lack of affordable services and wait lists were common experiences for service users. They felt this lack of services affected their MSD(V) issues adversely and caused extra suffering. Participants indicated that timely support was particularly important in relation to substance use: “when you have an addiction…[you think] I’m going to get some help, and then when you…[can only] make an appointment 2 months from now—a lot changes within the 2 months and by then you don’t care anymore” (SU).

To reduce wait times and improve access to appropriate services, participants recommended a greater number of local MSD(V) services, especially in the rural community: “more psychiatrists should be in [this area]…right now…you are looking at [a wait of] 6 months. Now what are you supposed to do in the interim?” (FM); “I think [the rural location’s] ER needs a mental health section…They have [a psychiatrist]…and she is the only shrink in the county…the closest detox is in [the city]…there’s no resources for someone that’s got any drug addiction or anything” (SU).

Participants also suggested that a broader range of programming for MSD(V) issues be covered by provincial health insurance: “you have to wait if you want to go to a psychiatrist. I like my psychologist. Problem with psychologists is they are not covered by [Ontario medicare]…and a lot of people can’t afford…to go to a psychologist” (SU); “I personally think that it should be a part of our health care. It’s an illness…I didn’t wake up one day and say you know what I think I’m going to start smoking crack today. It didn’t happen like that” (SU).

##### Early intervention

Based on their experiences, participants recommended that supports for MSD(V) issues be provided before they become severe or require more intensive care: *“*it shouldn’t have to be an emergency situation before you can get assigned to a psychiatrist…I knew people who have been on a list for years…my recommendation was you go to [the Emergency Department]…it’s the only way you’re gonna get…a psychiatrist or a diagnosis for that matter” (FM).

Because most service users experienced issues during childhood, they also emphasized the importance of addressing MSD(V) issues among youth: “the main thing that changed me was the treatment that I got when I was young…but if you don’t get that treatment when you’re young, you never learn how to cope with [mental health issues]” (SU).

#### System issues specifically of concern to family members

As shown in Table 4, two additional system-related themes emerged from interviews with family members that were not mentioned by service users, greater involvement of family members and mechanisms for treatment compliance.

##### A system that supports greater involvement of family members

Some family members were frustrated by the lack of support for family members who are coping with an individual with MSD(V) issues: “each time that [my brother] visited one of the hospitals, we were kind of begging them, pleading for them to please admit him…and then direct us somehow on what we can do because we didn’t know what to do” (FM). Others felt that family members were not included in the treatment process, often due to privacy laws: “the way the system’s set up is that—here is a person with a total irrational mind, but he’s the one that can make all the decisions for himself, and there’s no input allowed from the family if he doesn’t want that input to be given—so it’s just a crazy system for crazy people” (FM).

Because of their extensive knowledge of what was happening with their relative and their ultimate responsibility for the service user, some family members suggested that help for the service user could be improved if the family member’s insights were taken into consideration in treatment planning: “I think there should be a way to involve all the relevant family members…setting up any kind of a program for a person with mental illness or substance abuse problems, because it’s not an individual problem, it’s a family problem” (FM).

##### Mechanisms for treatment compliance

Several family members expressed frustration at a perceived lack of options in situations where they recognized that their relative needed help for MSD(V) issues, but the service user did not recognize the issues or was not ready to seek help: “I was in the phone book calling all the mental health and stuff like that, asking those people for help for her, but nobody would help her. Nobody would help me either…they are like ‘she has to do it herself, she has to come to us and we can’t do anything for you’” (FM). Some of these family members wanted a mechanism to compel people with MSD(V) issues to take medications and to participate in treatment: “I’m enraged that as a society we are giving money to people with a mental illness and making no requirements of them to better themselves. We’re just throwing money at the problem…to get them off the street and out of everybody’s visibility” (FM). Rather than mandating treatment, other family members believed that consistent outreach and education might help people to recognize their MSD(V) issues and agree to treatment: “maybe if they actually stepped out and came to visit her like 4 or 5 times she would of went, ‘you know, I do need help’” (FM).

## Discussion

The aim of this study was to describe the perspectives of those who have sought help for mental health, substance use and/or violence MSD(V) issues (i.e., “service users”), as well as their family members. This research is novel in its inclusion of a large heterogeneous sample of both service users and family members who described their experiences getting help for a broad range of single and co-occurring MSD(V) issues. A substantial proportion (40 %) of the sample had experienced all three issues, providing insight into the experiences of those with multiple issues that has not been addressed by previous research. Through participants’ experiences and recommendations, we were able to glean important information needed for planning system improvements for persons with MSD(V) issues. Other researchers [37] have noted that individuals who use mental health services are heterogeneous in their issues and needs; this appears to be even more evident for service users with co-occurring issues. Thus, a variety of services are needed to address the diverse needs of MSD(V) service users as well as coordination between these services.

A significant desired treatment outcome, expressed by both service users and family members, was for the service users to feel and be “normal”; this goal also permeated discussions about experiences with and recommendations for services. A desire to be “normal” is partly about problem remediation but also reflects typical life expectations, reflecting recovery-oriented goals [19], including self-reliance, independence, empowerment and having choices. Feeling “normal” also relates to one’s ability to feel connected to society [16] and to live a “satisfying, hopeful, and contributing life even within the limitations caused by illness” [38, pp 354]. Thus, for people with chronic MSD(V) issues, service providers and informal supports need to help service users and family members move forward from the grief caused by the disorder, to accepting and coping with their current issue, finding new meaning in life, and adapting in ways that will help them feel they are able to lead a more “normal” life that includes feeling connected to others and working towards goals and aspirations [18, 39, 40]. The themes that emerged under desired outcomes of a better understanding of their MSD(V) challenges and how to manage them, addressing practical needs and improving social relationships are all part of this broader recovery process.

Regardless of the service from which they sought help, participants identified several characteristics as important components of programming for MSD(V) issues. As with earlier studies [20, 24, 41, 42], participants wanted to be treated respectfully and listened to by service providers. Consistent with findings from research on service user experiences with mental health [43, 44] and substance use services [21], participants attributed experiences of disrespect to service providers’ ignorance about MSD(V) issues, stigma and stereotypes. To reduce stigma and misinformation, participants recommended more widespread education and outreach that might highlight the prevalence of MSD(V) issues, including co-occurring issues, promote local resources addressing these issues, and facilitate early intervention.

Consistent with previous research showing benefits of peer support and education for MSD(V) issues [45], some participants preferred services that provided peer support and professional staff with lived experience, in part, because they helped to normalize experiences with MSD(V) issues and provided hope for recovery. Additionally, as found in previous research [44], there were mixed views on the use of medications. Whereas some participants described medications as essential to their recovery, others felt medications were over-prescribed or provided when other forms of help were needed instead. These differences might partly reflect differing needs related to specific disorders; however, they also reflect participants’ concerns that medications were not always in their best interests, that the prescription of medications without counselling was not sufficient, or that physicians need to hear and respond to service user questions and concerns about specific medications or dosages.

Most participants expressed the need for services where there is someone they can talk to. Related to this, some participants identified the need for counselling services from psychologists and other non-medical professionals to be publicly funded rather than paid for by users (to supplement services provided by psychiatrists). These findings are consistent with previous recommendations for improving access to supportive counselling and providing services that assist service users in fostering a sense of purpose and connections with others and address practical needs associated with MSD(V) issues (e.g., housing and employment) [46, 47]. Assisting service users with practical needs can also help to lessen the “burden of care” for family members [48].

Despite research with similar calls to action more than a decade ago [26, 49], the present findings suggest that effective integrated care is still the exception. Service providers are increasingly recognizing the connections among MSD(V) issues [14] and the need for services to address co-occurring disorders [50–52]; nevertheless, navigating a system with specialized and discrete services was identified as an ongoing challenge for service users, especially for people with co-occurring MSD(V) issues. Our findings support calls for better coordination between medical and community services, such as the development of community health centres and other models of collaborative care [see also 53–57] as well as funding for nonmedical counselling services [see 58].

Although many of the same concerns were raised by service users and family members, some important differences were also identified. Family members experienced frustration with a system that excludes them from treatment decisions and with the general lack of information and support for family members. Balancing the rights of service users with family members’ needs for information and support is challenging [59]. The desires expressed by family members’ for more input and control, and particularly the suggestion that service users be forced to comply with treatment, are in direct contradiction to recent recovery-oriented approaches that emphasize the critical role of agency and self-determination on the part of the service user [18]. These desires stem from a concern that service users may not always be capable of acting in their own best interests. Research suggests that it may be possible for family members to have a role in service coordination [30], support the recovery process and supplement the work of formal services [29, 43] and have a positive impact on care [60]. However, if service users are to be directors of their own care, as articulated in the recovery approach, both service providers and family members may need to accept and adapt to this model of care. This is a critical area for further research.

## Limitations

Most (82.9 %) family member participants in our sample were female, possibly reflecting that the majority of informal caregivers to people with chronic health conditions are female [61]. Also, the sample for this study was comprised of participants from two communities in Southwestern Ontario, Canada. Although many of the issues raised by participants echo those of service users in previous research, experiences may be somewhat different in other geographic areas with different systems of services.

## Conclusion

Recommendations by service users and family members reinforce current efforts to better align the system of care with the needs of service users and their families [62]. New strategies need to be developed to support the caregiving efforts of families while respecting the rights and autonomy of service users. Building on these findings, future research needs to address more systematically commonalities and differences in perspectives of those with single versus multiple MSD(V) issues and to explore evidence-based collaborative models of care for MSD(V) issues.
